# FRED flow diversion with LVIS protection of large posterior communicating artery aneurysm: the "FRELVIS" technique

**DOI:** 10.3171/2022.7.FOCVID2262

**Published:** 2022-10-01

**Authors:** Steven B. Housley, Justin M. Cappuzzo, Muhammad Waqas, Andre Monteiro, Elad I. Levy, Adnan H. Siddiqui

**Affiliations:** 1 Department of Neurosurgery, Jacobs School of Medicine and Biomedical Sciences, University at Buffalo;; 2 Department of Neurosurgery, Buffalo General Medical Center, Kaleida Health, Buffalo;; 3 Department of Radiology, Jacobs School of Medicine and Biomedical Sciences, University at Buffalo;; 4 Canon Stroke and Vascular Research Center, University at Buffalo; and; 5 Jacobs Institute, Buffalo, New York

**Keywords:** Flow Redirection Endoluminal Device, FRED, low-profile visualized intraluminal support, LVIS, flow diverter, posterior communicating artery aneurysm

## Abstract

Treatment of wide-necked posterior communicating artery (PCoA) aneurysms is extremely challenging, especially in fetal posterior cerebral artery (PCA) configurations. This technical video demonstrates the nuances of an innovative use of flow diversion to treat a recurrent wide-necked PCoA aneurysm. This middle-aged patient presented with recurrence of a previously ruptured, coil-embolized PCoA aneurysm. Initial attempts at Comaneci-assisted coiling were unsuccessful because the coil herniated into the middle cerebral artery (MCA). Therefore, a low-profile visualized intraluminal support (LVIS) was placed in the fetal PCA across the aneurysm ostium and a flow diverter was placed in the internal carotid artery and MCA to constitute a Y-construct.

The video can be found here: https://stream.cadmore.media/r10.3171/2022.7.FOCVID2262

## Transcript

### 0:27 Patient’s History and Imaging Findings.

This is a case of FRED flow diversion with LVIS protection for a large posterior communicating artery aneurysm—the FRELVIS technique—in an elderly woman who was found down at home and transported to the ED, where she was found to have a Hunt-Hess 3, Fisher 4 subarachnoid hemorrhage on noninvasive imaging, and an irregularly shaped right posterior communicating artery aneurysm was observed and was subsequently coil embolized.^1^

### 0:42 CT and CTA 3D Reconstructions.

A follow-up angiography demonstrated a recurrent neck, and a decision was made to pursue additional treatment. Here is the initial CT showing the Fisher 4 subarachnoid hemorrhage as well as the CTA reconstructions demonstrating the aneurysm.

### 0:54 Reconstructed 3D DSA Imaging Showing the Aneurysm Prior to First Treatment.

Here are 3D DSA images showing the initial aneurysm prior to first coil embolization.

### 1:04 DSA Follow-Up Demonstrating Recurrence.

Here are follow-up DSA images showing the recurrent neck of the aneurysm following coil compaction.

### 1:14 Internal Carotid Artery Access.

Radial access was obtained and the Armadillo guide catheter was brought over the 0.035 Glidewire into the right common carotid artery. This is demonstrated here. At this point, you can see the Armadillo guide catheter preparing to make the bend into the common carotid artery. We are able to advance the Armadillo guide catheter into the internal carotid artery.

### 2:09 Selective Catheterization of the Posterior Communicating Artery (DSA AP/LAT).

At this point, a Synchro Select microwire was used to access the right posterior communicating artery in preparation for placement of the LVIS stent across the orifice.^2^ Here you can see this fetal-type posterior communicating artery, and this artery is relatively large in diameter and therefore supplying significant flow to the ipsilateral posterior cerebral artery.

### 2:45 Temporary Coil Deployment (DSA AP/LAT).

Once our microcatheter is in place, we place a second microcatheter into the aneurysm and place a coil partially in to help lock that catheter into place.

### 2:58 LVIS Jr. Deployment (DSA AP/LAT).

The LVIS Jr. 2.5 × 23–mm stent is then brought in the posterior communicating artery and deployed. This is used to ensure integrity of the orifice of the posterior communicating artery.^3^

### 3:18 Comaneci Attempt.

We then make a quick attempt at using a Comaneci device to coil the aneurysm. However, this was not possible secondary to the largest Comaneci device available not being large enough to cover the aneurysmal orifice.^4^

### 3:30 FRED Deployment.

Therefore, a 4.5 × 20–mm FRED flow diverter was placed across the orifice of the aneurysm as well as the posterior communicating artery through the internal carotid artery.^5,6^

### 3:43 Final Runs and Contrast Stasis.

Here is our final run demonstrating stasis of contrast within the aneurysm as well as the final construct.

### 3:54 Clinical Outcome.

The patient tolerated the procedure well and was discharged home on postoperative day 1 at her neurological baseline.

### 3:58 MRA Follow-Up.

At approximately 3 months postprocedure, an MRA was obtained which shows delayed but continuous filling of the aneurysm. Here you can see multiple images demonstrating this. This is not unexpected with flow diversion.^7^

### 4:11 Planned Follow-Up.

The patient will continue to be followed closely for possible recurrence and/or resolution.

## Disclosures

Dr. Cappuzzo: consulting fees from Cerenovus, J&J Medical Device Companies, Integra Lifesciences Corp., MIVI Neuroscience Inc., Penumbra Inc., and Stryker Corp.; and support for attending meetings and/or travel from Stryker Corp. and Penumbra Inc. Dr. Levy: shareholder/ownership interest in NeXtGen Biologics, RAPID Medical, Claret Medical, Cognition Medical, Imperative Care, Rebound Therapeutics, StimMed, and Three Rivers Medical; patent with Bone Scalpel; honorarium for training and lectures from Medtronic, Penumbra, MicroVention, and Integra; consultant for Clarion, GLG Consulting, Guidepoint Global, Imperative Care, Medtronic, StimMed, Misionix, and Mosiac; chief medical officer for Haniva Technology; national PI for Medtronic—Steering Committees for SWIFT PRIME and SWIFT DIRECT Trials; site PI for MicroVention (CONFIDENCE Study) and Medtronic (STRATIS Study-Sub 1); advisory board for Stryker (AIS Clinical Advisory Board), NeXtGen Biologics, MEDX, Cognition Medical, Endostream Medical, and IRRAS AB (Consultant/Advisory Board); personal fees for rendering medical/legal opinions as an expert witness; and leadership or fiduciary roles in CNS, ABNS, and UBNS. Dr. Siddiqui: consulting fees from Amnis Therapeutics, Apellis Pharmaceuticals Inc., Boston Scientific, Canon Medical Systems USA Inc., Cardinal Health 200, LLC, Cerebrotech Medical Systems Inc., Cerenovus, Cerevatech Medical Inc., Cordis, Corindus Inc., Endostream Medical Ltd., Imperative Care, InspireMD Ltd., Integra, IRRAS AB, Medtronic, MicroVention, Minnetronix Neuro Inc., Peijia Medical, Penumbra, Q’Apel Medical Inc., Rapid Medical, Serenity Medical Inc., Silk Road Medical, StimMed, LLC, Stryker Neurovascular, Three Rivers Medical Inc., VasSol, and Viz.ai Inc.; secretary of Board of the Society of NeuroInterventional Surgery (2020-2021); chair of Cerebrovascular Section of the AANS/CNS (2020-2021); stock or stock options in Adona Medical Inc., Amnis Therapeutics, Bend IT Technologies Ltd., BlinkTBI Inc., Cerebrotech Medical Systems Inc., Cerevatech Medical Inc., Cognition Medical, CVAID Ltd., E8 Inc., Endostream Medical Ltd., Galaxy Therapeutics Inc., Imperative Care Inc., InspireMD Ltd., Instylla Inc., International Medical Distribution Partners, Launch NY Inc., Neurolutions Inc., NeuroRadial Technologies Inc., NeuroTechnology Investors, Neurovascular Diagnostics Inc., Peijia Medical, PerFlow Medical Ltd., Q’Apel Medical Inc., QAS.ai Inc., Radical Catheter Technologies Inc., Rebound Therapeutics Corp. (purchased in 2019 by Integra Lifesciences Corp.), Rist Neurovascular Inc. (purchased in 2020 by Medtronic), Sense Diagnostics Inc., Serenity Medical Inc., Silk Road Medical, Sim & Cure, SongBird Therapy, Spinnaker Medical Inc., StimMed, LLC, Synchron Inc., Three Rivers Medical Inc., Truvic Medical Inc., Tulavi Therapeutics Inc., Vastrax, LLC, VICIS Inc., and Viseon Inc.: and national PI/steering committees for Cerenovus EXCELLENT and ARISE II Trial; Medtronic SWIFT PRIME, VANTAGE, EMBOLISE, and SWIFT DIRECT Trials; MicroVention FRED Trial and CONFIDENCE Study; MUSC POSITIVE Trial; Penumbra 3D Separator Trial, COMPASS Trial, and INVEST Trial; MIVI Neuroscience EVAQ Trial; Rapid Medical SUCCESS Trial; and InspireMD C-GUARDIANS IDE Pivotal Trial.

Devices included in this video and their manufacturers: Armadillo guide catheter, Q’Apel Medical; Comaneci device, Rapid Medical; FRED, MicroVention; LVIS Jr., MicroVention; and SynchroSelect microwire, Stryker Neurovascular.

## Author Contributions

Primary surgeon: Siddiqui. Assistant surgeon: Housley, Cappuzzo, Waqas. Editing and drafting the video and abstract: all authors. Critically revising the work: all authors. Reviewed submitted version of the work: all authors. Approved the final version of the work on behalf of all authors: Siddiqui. Supervision: Siddiqui, Cappuzzo, Levy.

## Supplemental Information

### Patient Informed Consent

The necessary patient informed consent was obtained in this study.
